# Biodiversity Sampling Using a Global Acoustic Approach: Contrasting Sites with Microendemics in New Caledonia

**DOI:** 10.1371/journal.pone.0065311

**Published:** 2013-05-29

**Authors:** Amandine Gasc, Jérôme Sueur, Sandrine Pavoine, Roseli Pellens, Philippe Grandcolas

**Affiliations:** 1 Département Systématique et Évolution, Muséum national d'Histoire naturelle, Paris, France; 2 Département Ecologie et Gestion de la Biodiversité, Muséum national d'Histoire naturelle, Paris, France; 3 Department of Zoology, University of Oxford, Oxford, United Kingdom; University of Kent, United Kingdom

## Abstract

New Caledonia is a Pacific island with a unique biodiversity showing an extreme microendemism. Many species distributions observed on this island are extremely restricted, localized to mountains or rivers making biodiversity evaluation and conservation a difficult task. A rapid biodiversity assessment method based on acoustics was recently proposed. This method could help to document the unique spatial structure observed in New Caledonia. Here, this method was applied in an attempt to reveal differences among three mountain sites (Mandjélia, Koghis and Aoupinié) with similar ecological features and species richness level, but with high beta diversity according to different microendemic assemblages. In each site, several local acoustic communities were sampled with audio recorders. An automatic acoustic sampling was run on these three sites for a period of 82 successive days. Acoustic properties of animal communities were analysed without any species identification. A frequency spectral complexity index (*NP*) was used as an estimate of the level of acoustic activity and a frequency spectral dissimilarity index (*D_f_*) assessed acoustic differences between pairs of recordings. As expected, the index *NP* did not reveal significant differences in the acoustic activity level between the three sites. However, the acoustic variability estimated by the index *D_f_*, could first be explained by changes in the acoustic communities along the 24-hour cycle and second by acoustic dissimilarities between the three sites. The results support the hypothesis that global acoustic analyses can detect acoustic differences between sites with similar species richness and similar ecological context, but with different species assemblages. This study also demonstrates that global acoustic methods applied at broad spatial and temporal scales could help to assess local biodiversity in the challenging context of microendemism. The method could be deployed over large areas, and could help to compare different sites and determine conservation priorities.

## Introduction

New Caledonia has been classified as one of the 25 most important hotspots of biodiversity conservation regarding the number of endemic species and degree of threat [1]. Kier et al. [2] also ranked New Caledonia as the terrestrial region with the highest level of endemism. As an example, 19% of bird, 67% of mammal, 86% of reptile and 76% of plant species are endemic to this Pacific island at the regional scale [1]. The emphasis put on regional endemism and biogeography masks another remarkable feature of New Caledonia, the extremely high level of local endemism, hereafter called microendemism. The so-called microendemic species of plants, lizards or insects show a very short distributional range, limited to small mountains or rivers of New Caledonia. According to recent studies, this local endemism mainly originated through recent allopatric speciation with little ecological differentiation [3], with the notable exception of adaptation to metalliferous soils derived from ultramafic rocks covering one third of the island [4]. In this paradigm, the island can be considered as a wonderful natural laboratory for evolution, where many questions regarding speciation and endemism can be studied through a large time window of 37 million years (e.g., [3], [5]–[8]).

Microendemism also bears strong consequences for conservation since very short distributional ranges increase the risk of species extinction, facing three principal threats that are nickel mining, fire and invasive species (reviewed in [9]). The origin and composition of this threatened biodiversity have to be quickly described and deciphered, both to allow theoretical studies of speciation and evolution to be conducted and to permit the establishment of conservation policies. It is therefore necessary to rapidly characterize sites and their communities. However, such a task is made complex because sites differ not only by distinct species combinations within the same local pool, but also by the fact that each mountain or river usually shows local endemics formed by allopatric speciation (e.g., [7]–[8], [10]). Inventorying in New Caledonia has proved to be a fastidious and slow process due to a complex landscape, a small local scientific community, and long distance isolation from most academic centres. The level of species richness in New Caledonia is particularly high throughout the island. Comparing species richness of sites is not likely to be very informative [9]. Establishing complementarities of different sites in terms of species composition should help in establishing conservation priorities for the diversity evaluation and conservation of New Caledonia [11]–[12].

Therefore, inventorying and evaluation processes need to be enhanced and speeded up by using other approaches. In this respect, inventories based on passive acoustic methods have demonstrated convincing advantages as they are non invasive, allow large automatic sampling, can be simultaneously used on several taxa, and provide very large temporal and spatial data sets [13]–[14]. Similar to classical inventory methods, passive acoustics can potentially be used to identify species directly by ear [15]–[16] or by automatic identification methods [17]–[20]. However, identification of singing species by human observers is partly subjective depending on the experience and listening skills of the observer and to the difficulty of handling large samples. Automatic identification processes, which aim at finding species-specific songs, require an important sound reference database. Such an approach seems very complex to undertake in New Caledonia where species richness is particularly high, and where species acoustic diversity has not yet been fully registered and described. Facing these difficulties, a global acoustic passive method might therefore be especially helpful to provide new data on local animal diversity [21].

The global acoustic method consists of analysing the acoustic output of animal communities by measuring how diverse or complex a community's signal is, in terms of spectral and/or temporal acoustic properties, without any species identification. Such measures of acoustic complexity have been linked previously to the number of singing species [21]–[22] and to the number of vocalizations [23]. Following Diamond and Case [24], who defined a community as an entity that “comprises the populations of some or all species coexisting at a site or in a region”, an acoustic community could be seen as a collection of sounds produced by all living organisms in a given habitat over a specified time. In this case, the species compete for the sound space, which is itself a sound resource. Two complementary approaches are currently developed for a rapid acoustic survey, one to evaluate the acoustic diversity level of each community based on a measure of acoustic signal complexity (α diversity), and the other to compute acoustic distances between pairs of communities (β diversity) [21].

The aim of this study is to evaluate the capacity of the global acoustic method to detect dissimilarity between sites that are otherwise known to be similar with respect both to species richness and ecological context but distinct regarding species composition and microendemics. To do this, three sites on the main island have been selected because of two characteristics: a high and comparable level of species richness and differences in species composition mainly due to microendemism. These sites were selected according to a census based on all published phylogenetic and inventory studies so far (see Table S1), and on Global Biodiversity Inventory Facility (GBIF) records from collection databases. Automatic recorders collected acoustic data on these three sites. Distance based acoustic indices and statistical analyses assessed the acoustic dissimilarities among the three different sites.

The study can be seen as a very stringent test of the capacity of the global acoustic method to detect dissimilarity of biodiversity, given that allopatric speciation with niche conservatism, which is important in New Caledonia, is expected to maximize ecological similarity between sites. According to this capacity to detect dissimilarity, the global acoustic method could be an effective and efficient new approach to contrast sites of high biodiversity value.

## Materials and Methods

### Study sites

**Figure 1:**
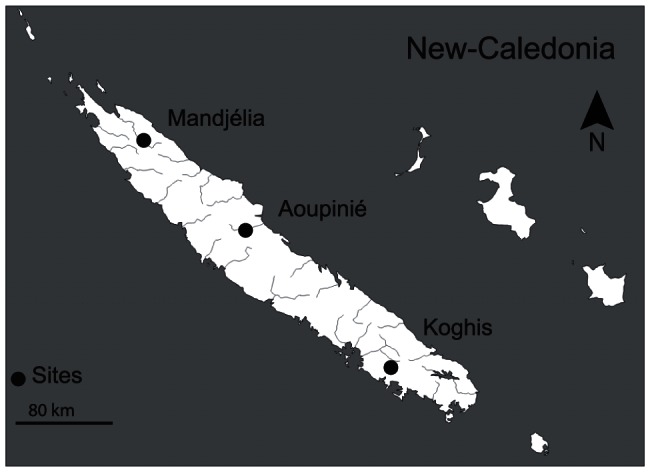
Sampled sites in the great island of New Caledonia. Three audio recorders were settled, each at a similar altitude of 600 m a.s.l. and each separated by 200 m.

Mandjélia (20°24.098′S, 164°31.458′E), Aoupinié (21°10.034′S, 165°19.017′E) and Koghis (22°10.833′S, 166°30.632′E) mounts were chosen as reference sites for their distinct species occurrence and specific microendemism (Figure 1). This selection was made in accordance with the most accurate inventories performed so far in the framework of 18 phylogenetic and 7 inventory studies published on varied groups of organisms in New Caledonia (see Table S1 in Supporting Information). These studies gathered 269 species and 33 genera including plant and animal species with only a few of them producing sound.

The Global Biodiversity Information Facility (GBIF, [25]) provided a dataset of all specimens collected on a square of 80 km^2^ around the three sites, belonging to both animal and vegetal taxa. The most accurate spatial scale available on the GBIF portal was chosen. The number of species was dependent on the collection effort that was not comparable from one site to another. In order to correct this bias, the number of species for each site was divided by the number of collectors. Other potential hidden bias, such as different sampling efforts and techniques, were not provided by the GBIF portal and could not be taken into account.

Climate (tropical humid), soil (non metalliferous soils) and vegetation (tropical humid forest) of the three sites were similar. Within each site, three recorders were placed at a distance of 200 meters from each other. The sample effort was increased by settling several recorders per site to reduce the spatial coverage difference between acoustic and inventories data. All recorders were placed at a similar altitude of 600 m (+/−84 m, n = 9).

### Passive acoustic recording

The recordings were made from 10 April to 30 June 2010 with Song Meter SM2 digital audio field recorders [26]. These offline and weatherproof recorders were equipped with a single omnidirectional microphone (frequency response: −35±4 dB between 20 Hz and 20 kHz) oriented horizontally at a height of 1.5 m. The signals were digitized at a sampling frequency of 44.1 kHz and a depth of 16 bits quantization. The files were saved in the lossless compressed format .*wac* and then transformed into the format .*wav* with the software WAC to WAV Converter Utility version 1.1 [26]. The first minute of every hour was recorded leading to a total of 13,602 sound files.

All recordings were examined by A.G. to remove files where the occurrences of rain, wind or anthropogenic noise were detected and could bias the results. This resulted in a selection of 6,571 files (53% for Aoupinié, 48% for Mandjélia and 49% for Koghis, see Table S2).

No specific permits were required for the described field studies because a passive acoustic method was used in an unprotected public location. This also implies that the study did not involve endangered or protected species. The acoustic recordings were deposited in the sound library of the Muséum national d'Histoire naturelle (Paris, France, contact: sonocollections@mnhn.fr).

### Acoustic activity level

A level of acoustic activity was defined as the number of song types and was assessed by ear by A.G. for each recording. A song type was identified as any unique acoustic sequence that can be differentiated by a human ear based on frequency and time-amplitude features.Three levels were used: (1) “Moderate to high activity” where more than two different song types were identified, (2) “Low activity” where one or two different song types occurred, and (3) “Null activity” where no animal songs could be detected (see Table S3). This scale was designed to fulfil aural discrimination constraints on a very large data set (6,571 files). In particular, it was not possible to clearly distinguish more than two song types in complex recordings. This characterisation of the acoustic activity level was also built for an easy use by other potential observers. The acoustic activity detected was mainly due to bird, Orthoptera and cicada species, but no species identification was achieved.

### Acoustic complexity index *NP*

A new index was developed to assess acoustic complexity. This index, named *NP* for “Number of Peaks”, counts the number of major frequency peaks obtained on a mean spectrum scaled between 0 and 1. A mean spectrum was obtained for each audio file by computing a short-time Fourier transform (STFT, non overlapping window size = 512 samples = 11 ms). A specific function was developed within the ‘seewave’ package [27] of the R environment [28] to process the automatic detection of frequency spectral peaks of each mean spectrum. All peaks of the spectrum were first detected and then selected using amplitude and frequency thresholds. The first selection factor was based on the amplitude slopes of each peak. Only peaks with slopes higher than 0.01 were kept. The second selection factor was based on frequency. In cases where consecutive peaks were less than 200 Hz apart, only the highest peak in amplitude was kept. *NP* was simply defined as the number of peaks after detection and selection of peaks.

### Acoustic dissimilarity index

Global differences were measured using the acoustic frequency spectral dissimilarity index, named *D_f_* for “Dissimilarity of frequencies”, as defined by Sueur et al. [21]. This index has been shown to be sensitive to the diversity of the community assemblages information [29]. *D_f_* was computed according to:
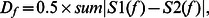
where S1(*f*)and S2(*f*) are the probability mass functions of the mean spectra of the two recordings to be compared. Each function was the result of a STFT with a non overlapping window of 512 samples ( = 11 ms).

*D_f_* was assessed between all pairs of audio files leading to a dissimilarity matrix. Each recording was associated with a recording time, a day, a recorder, and a site. To factor out the differences between days with different weather conditions, the differences between each recording were averaged over all available days leading to a matrix with dimensions 216*216 ( = (3 sites * 3 recorders * 24 time periods ) * (3 sites * 3 recorders * 24 time periods)). Then, the matrix was transformed by the Lingoes approach to reach Euclidean properties [30].

### Background noise reduction

A preliminary analysis was conducted to test whether differences between pairs of recordings could be due to different background noises and not to different biotic sounds. The dissimilarity matrix obtained solely with recordings with background noise (Null activity) was analyzed with a Principal Coordinate Analysis (PCoA) [31] in order to visualize whether the factors “Site” (Figure S1A) and “Time” (Figure S1B) could explain the acoustic variability, and to identify which factor was the most important in explaining the acoustic differences observed. Each point projected in the resulting multidimensional space represented one recorder at one recording time. The role of the “Site” and “Time” factors in explaining the acoustic differences between sites due to background noise was evaluated with a distance-based Redundancy Analysis (dbRDA) [32] applied on the acoustic dissimilarity matrix. Associated to the dbRDA, a permutation test was applied with 1,000 permutations considering each factor independently. This ensured that the permutations used to test the factor “Site”, were constrained by the factor “Time” and *vice versa*. The dbRDA results highlighted differences between sites when considering only files with noise (p = 0.001, Figure S1C). To check that these differences between sites were not due to time differences, the dbRDA associated to the permutation test was also applied with the factor “Time” (p = 0.437, Figure S1D). These preliminary results led to the application of a filtering process on all files to remove the noise associated to the sites as much as possible before calculating the acoustic indices. For each site, all recordings with null activity were selected and averaged to obtain the mean spectrum of ambient noise that was computed using a STFT based on a non-overlapping sliding function window of 512 samples ( = 11 ms). The mean spectrum of each file was subtracted by the mean spectrum of ambient noise of the local site. This subtraction was weighted by the amplitude level of each recording as follows:
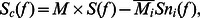
where *S_c_(f)* is the probability mass function of the mean spectrum corrected, *S(f)* is the probability mass function of the original mean spectrum, *Sn_i_*(*f*) is the probability mass function of the mean spectrum of the ambient noise of the site *i*, *M* the amplitude level of the recording and 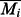 the mean of amplitude levels of files containing ambient noise for the site *i*. The amplitude level parameter *M* was obtained following:
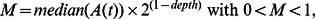
where *A*(*t*) is the amplitude envelope and *depth* is signal quantization (here, 16 bits). Only the files with “Moderate to high activity” and “Low activity” were kept for subsequent analyses.

### Statistical analyses

In order to evaluate the reliability of *NP* in revealing the acoustic activity detected by ear, the distribution of *NP* values measured before ambient noise reduction were compared between pairwise groups of activity level (moderate to high activity, low activity, null activity) with a non parametric Mann-Whitney test with a Holm correction for multiple tests. The same procedure was carried out to test the differences between *NP* values among sites, measured on spectra after ambient noise reduction.

The two factors “Site” and “Time” were considered to explain the acoustic variability contained in the dissimilarity matrix values. First, a PCoA and then, a dbRDA permutation test (1000 permutations) considering each factor independently, were applied as explained above (see Background reduction section). In order to quantify the acoustic differences between each pair of sites, the Euclidean distances of the barycentre of the points associated to the three levels of the factor “Site” were calculated, which corresponds to Rao's disc coefficient of dissimilarity between sites [33]–[34].

A last step was made to validate the PCoA and dbRDA results considering the factor “Site”. To ensure that these results were not biased by an unbalanced number of recordings per hour for each site, due to recordings being discarded because of bad weather conditions, the analyses were done a second time on a balanced subsample of hours from the averaged dissimilarity matrix. Six hours (1, 3, 4, 18, 20 and 21 h) with balanced samples were selected. The dimension of this submatrix was 54*54 ( = (3 sites * 3 recorders * 6 time periods) * (3 sites * 3 recorders * 6 time periods)). This submatrix was analysed with a dbRDA with site as a factor. The results were compared with the complete dissimilarity matrix with all hours included.

A final analysis was performed to detail the frequency spectral differences between the three sites. The dbRDA was used to potentially highlight acoustic differences between sites but was considered inappropriate to provide details regarding these differences. A specific analysis was developed to identify the frequency bins where differences occur. As already described, the mean spectrum was composed of 256 frequency bins and the *D_f_* index was the sum of these 256 differences between two mean spectra. Here, the 256 frequency bin differences calculated were not summed up but analysed independently. Instead of a single *D_f_* matrix, 256 matrices were analysed, one for each frequency bin. For each frequency bin, a between-site distance value was computed with the Rao's disc coefficient from the 256 matrices. This between-site distance was compared with the distribution of frequency peaks obtained for all sites.

All statistical analyses were performed with the R software including the package ‘ade4’ [35].

## Results

### Phylogenetic and GBIF data

From the 18 phylogenetic and 7 inventory datasets analysed, the percentage of microendemics calculated was 12.50% for Mandjélia, 15.38% for Aoupinié and 23.73% for Koghis. From the GBIF data , the ratio of endemic species to the number of collectors was 4.89 for Mandjélia, 3.76 for Aoupinié and 4.71 for Koghis.

### Acoustic activity level

**Figure 2:**
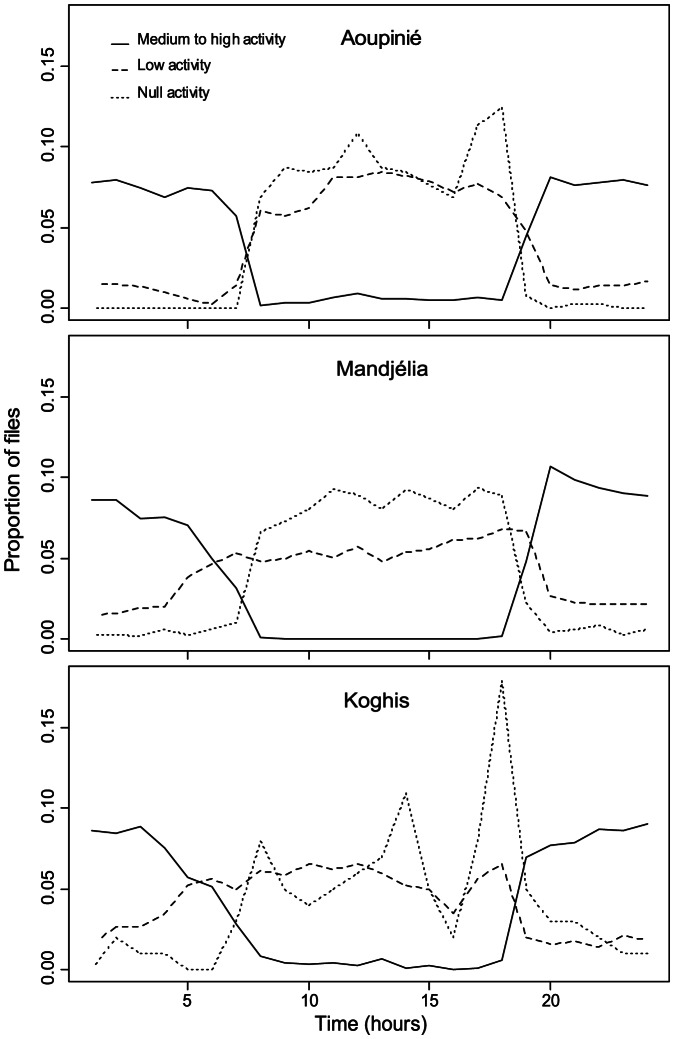
Acoustic activity on the three sites. Evolution of the proportion of files of moderate to high, low or null activity along the 24-hour cycle, for the Mandjélia, Aoupinié and Koghis sites. The sum of file proportions along the 24-hour cycle is equal to 1.

The acoustic activity level determined by ear showed variations along the 24-hour cycle (Figure 2). The recordings with high activity mainly occurred from 19 h to 7 h, whereas files with low and medium activity mainly occurred from 7 h to 18 h.

**Figure 3:**
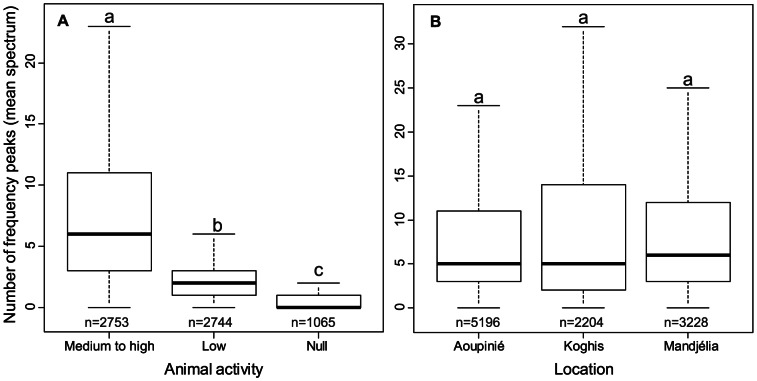
Number of frequency peaks. **A**: Boxplot of the number of frequency peaks measured on the mean spectrum of all recordings according to the activity level (Medium to High, Low or Null) assessed by ear (A.G.). **B**: Boxplot of the number of peaks measured on the mean spectrum of the recordings after noise reduction according to the sites. Similar letters above boxplots indicate no significant differences found by the Mann-Whitney test corrected with Holm correction.

The comparison between the level of animal activity determined by ear and the number of frequency peaks measured on the mean spectrum before ambient noise reduction highlighted an increase of *NP* with the level of animal activity (p<0.0001, Figure 3A). *NP* did not differ significantly between the three sites (Aoupinié-Mandjélia: p = 0.96; Aoupinié-Koghis: p = 0.17; Mandjélia-Koghis: p = 0.57, Figure 3B).

### Acoustic dissimilarity

**Figure 4:**
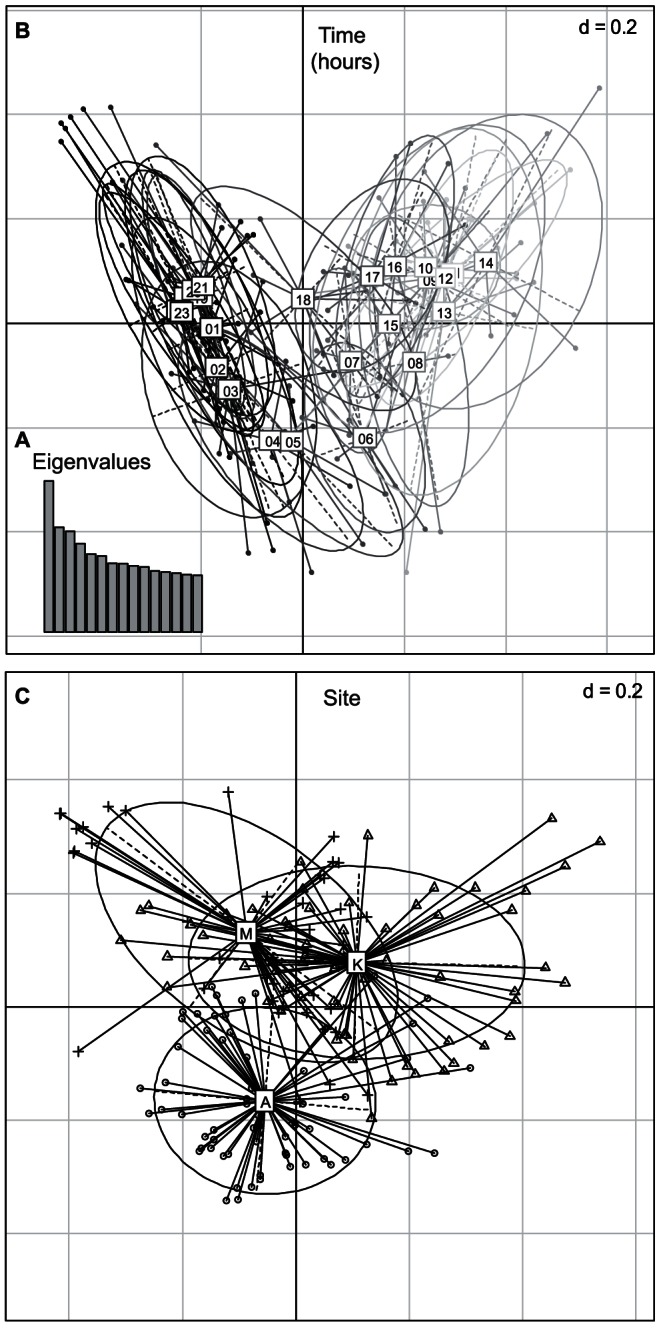
Principal Coordinate Analysis (PCoA) on the acoustic dissimilarity matrix. **A**: Eigenvalue bar plot. **B**: Results of PCoA applied to the acoustic dissimilarities among recordings with factor “Time” as a supplementary variable subsequently projected on the map. Projection along the axes 1 and 2. **C**: Results of PCoA applied to the acoustic dissimilarities among recordings with the spatial factor “Site” (A: Aoupinié, K: Koghis, M: Mandjélia). Projection along the axes 3 and 4. The dispersion ellipses surround the position of a time period (Figure 4A) or of a site (Figure 4B) providing an index of the dispersion around the time/site centroid (67% of recordings collected at a given time or at a site are expected to be in the associated ellipse).

The PCoA showed that the acoustic variability could first be explained by the factor “Time”, and then by the factor “Site”. The acoustic variability explained by the two first axes was associated with the factor “Time” (Figure 4A; axis 1: 1.92% and axis 2: 1.33% of variability explained). The first axis separated the night and day periods, whereas the second axis separated the middle of the day from the remaining hours of the 24-hour cycle. Hours were organized successively around the 24-hour rhythm. Axes 3 and 4 explained the variability due to the factor “Site” (Figure 4B; axis 3: 1.28% and axis 4: 1.12% of variability explained), independent of the variability already explained by axes 1 and 2. Mandjélia and Koghis appeared closer to each other than to Aoupinié as shown by a major overlap of the inertia ellipses. However, the rather flat distribution of eigenvalues indicated that the first four axes do not explain the entire variability.

**Figure 5:**
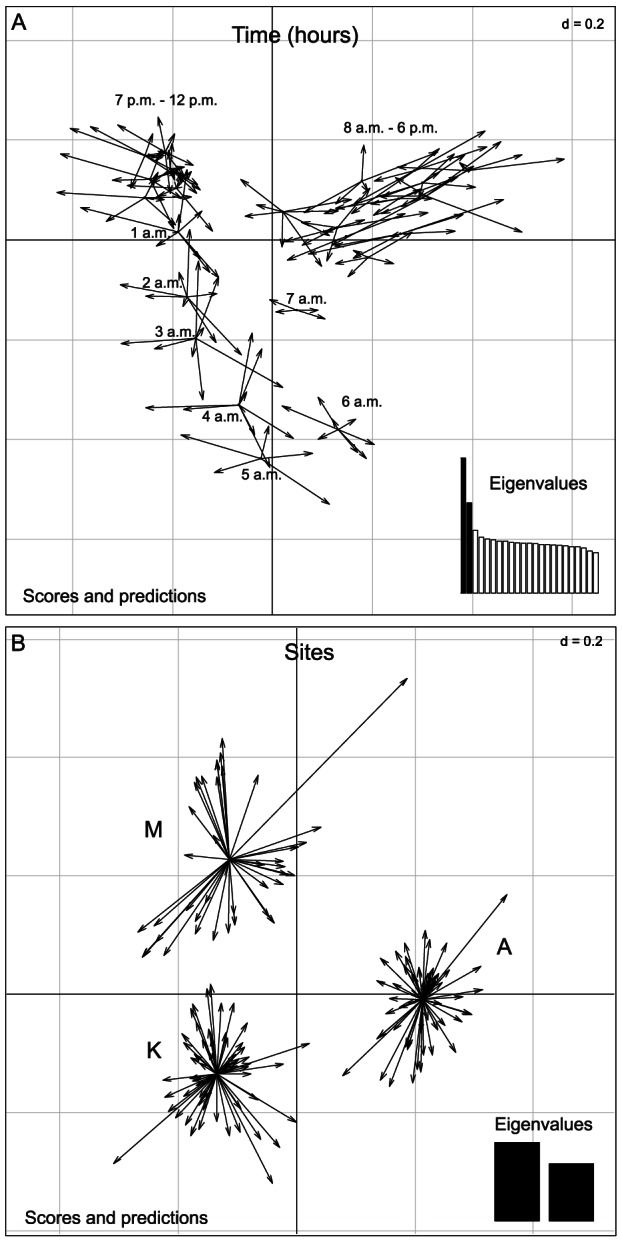
Distance-based ReDundancy Analysis (dbRDA). **A**: Results of dbRDA applied to the acoustic distances among recordings, with factor “Time” as an explanatory variable. **B**: Results of dbRDA applied to the acoustic distances among recordings, with factor “Site” as an explanatory variable (A: Aoupinié, K: Koghis, M: Mandjélia). The length of the arrows represents residuals: each arrow connects the position of a recording predicted by the time in which it was done or the site in which it was done (where the arrow starts) to its real position based on raw data (real acoustic composition where the arrow ends).

This approach was thus complemented by the dbRDA, which was first applied with the factor “Time” and then with the factor “Site”. This analysis validated the fact that the two factors could explain the acoustic distances variability (effect of difference among hours R^2^ = 0.852 for the first axis, R^2^ = 0.911 for second axis, and effect of differences among sites R^2^ = 0.863 for the first axis and R^2^ = 0.726 for the second axis; the third and fourth axes were independent of the first and second axes displaying temporal tendencies). Both axes fully discriminate the recording samples as a function of “Time” (Figure 5A) and “Site” (Figure 5B). The sites and hours were significantly different as shown by the dbRDA tests (p = 0.001 for both). A dbRDA was computed on a subset of the original data consisting of balanced samples. This dbRDA showed a similar full discrimination of sites (p = 0.001, see Figure S2) indicating that site discrimination, observed on the original matrix (216*216, Figure 5B), could not be due to unbalanced samples. Rao's coefficient of dissimilarity was 0.36 between Aoupinié and Mandjélia, 0.36 between Aoupinié and Koghis, and 0.33 between Mandjélia and Koghis. These results indicate that the three sites were acoustically distinct and were roughly at even acoustic distances.

**Figure 6:**
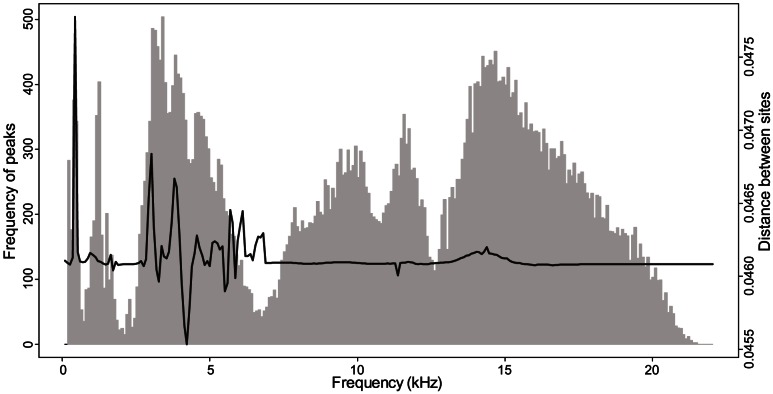
Histogram of the frequency used among all recordings. For each frequency, the value is the number of times that this frequency appeared as a peak among the mean spectra. The line is the difference measured between sites for each frequency.

The distribution of the frequency peaks detected on all recordings covered almost all the frequency range sampled, from 0.043 to 22.05 kHz (Figure 6). The distribution showed six main modes, around 0.5 kHz, 1.3 kHz, 4 kHz, 10 kHz, 11.5 kHz and 14.5 kHz respectively (Figure 6, grey histogram). The between-site distance based on the D*_f_* index showed important variations corresponding to the first three modes of the frequency peak distribution, i.e. around 0.5 kHz, 1.3 kHz, and 4 kHz (Figure 6, plain line).

## Discussion and Conclusions

Biodiversity distribution can be considered at nested temporal and spatial scales, from a broad (e.g. continental) to a narrow (e.g. a local altitudinal transect) scale [36]. However, describing such patterns requires the collection of considerable field-based data sets. These data are especially difficult to obtain in regions that are megadiverse and where species are under severe extinction threat, the so-called biodiversity hotspots [1]–[2]. From this point of view, New Caledonia shows an extremely high level of species richness and microendemism that is not yet sufficiently understood for efficient conservation efforts [3]. In such rich ecosystems, all local research on biodiversity from evolutionary biology to ecosystem management relies on classical species inventories that are time consuming and constrained by the availability of taxonomic expertise [9].

Recent ecological sensing methods should help in drawing such general diversity patterns by relaxing the constraint of taxonomic inventorying. In particular, non-invasive global acoustic approaches may be considered as an alternative or a complementary approach to classical biodiversity surveys [21], [37]. Comparing the sound properties of animal assemblages through the computation of acoustic dissimilarities may help in pointing out differences between sites that have distinct species composition, here partly related to different evolutionary histories and microendemism. A preliminary test for the efficiency of such an approach was run here on three major diversity sites in New Caledonia. Global acoustic analysis discriminated sites that are known for their high and comparable level of species richness, according to records in biodiversity meta-databases GBIF [25], and for their high level of microendemism according to all reported phylogenetic studies.

Acoustics is an emerging route for broad scale diversity estimation and as such has produced promising indices: a temporal and frequency spectral entropy index (index *H*, [21]), a ranked index combining signal amplitude and temporal entropy (index *AR*, [22]), and a spectrogram complexity index (index *ACI*, [23]). The *H* index is easy to compute, but might be biased by unwanted background noise, the *AR* index probably misses important frequency spectral information by considering only amplitude and time scales, and the *ACI* is not sensitive to constant acoustic signals that may occur in tropical environments where cicadas and Orthoptera [38] produce sustained sounds. A research effort is therefore needed to compare and improve these indices. A new index, *NP*, based on the frequency spectral properties was developed here as a simple measure of frequency spectral complexity. *NP* is less sensitive to ambient noise and would appear to be linked to animal sound activity level, defined here as a number of different song types. As expected, this index returned similar values for the three sampled sites that have close species richness levels. Similar peak richness ensures that subsequent dissimilarity analyses through acoustic distances could be interpreted in terms of differences in the acoustic frequency spectral profile and not as a simple scale effect related to a different acoustic activity level. To go further, the ability of the *NP* index to estimate precisely the number of song types needs to be further explored. Additional research should focus on the new methods that could help to estimate accurately the number of song types in such rich acoustic environments. The identification of songs coming from several species that generate a complex acoustic recording will certainly open new research fields in bioacoustics and ecology.

Considering the temporal scale of acoustic activity on the three sites, acoustic activity level profiles (*NP*) during day and multivariate analyses based on pairwise frequency spectral distances (*D_f_*) clearly showed that acoustic variability among sites could first be explained by a 24-hour pattern. The distribution of the recordings within multivariate spaces (PCoA, dbRDA) suggests a structured succession of different acoustic communities. Such temporal organization is well known in both temperate and tropical environments with clear temporal windows occupied by specific groups [39]–[40], the best-studied periods being the dawn and dusk bird choruses [41]. Such temporal separation could be the outcome of acoustic inter-specific interference [42] or the result of different predator pressures according to the time of day [43]. As no attempt was made here to identify the singing species, the details of the successive groups along the day and night cycle could not be estimated. However, it was obvious that night acoustic communities were dominated by insects, mainly Orthoptera, whereas day acoustic communities were composed of birds and insects such as cicadas. Even if the acoustic level here was significantly higher during the night than during the day, sampling should not be restricted to the night period as information during low activity periods could also be significant and provide information regarding dissimilarity between sites.

The high temporal variability along the 24-hour cycle overcame the spatial variability that was first targeted. However, the three sites undoubtedly appeared distinct when projecting the samples on the third and fourth axes of the multivariate analysis (dbRDA results). The rapid acoustic survey seems to be sensitive enough to highlight differences among three sites with similar habitats and specific richness. These differences were not due to residual background noise or unbalanced time sampling but were associated with differences in animal community sounds. Besides, similar *NP* values for each site ensured that differences were not due to different activity levels, but to different acoustic community compositions. The accompanying data reporting the presence/absence of species in the three sites (phylogenetic data, GBIF data and other inventory data) were unfortunately not based on standardized sampling protocols, but nevertheless support the occurrence of important differences between the species assemblages, a strong part of which could be related to allopatric speciation between mountains and the establishment of subsequent microendemism [3]. The global acoustic approach returns a single distance value, but does not provide accurate information on the acoustic differences. Deciphering the patterns observed requires identifying the bands of the spectrum that differ among sites. The original analysis comparing the frequency peaks detected and the distance values between sites for each frequency revealed that most of the acoustic differences were occurring over a sharp frequency band below 7 kHz. This 7 kHz limit might be peculiar to our study and should not be blindly considered as a reference threshold for other studies. Interpreting these acoustic differences in details would require a perfect and exhaustive knowledge of singing species which is unfortunately outside the scope of the present study and actually highly difficult to reach in such a megadiverse region. Numerous insect and bird species endemic to New Caledonia can produce sound but the *Agnotecous* cricket genus was the single microendemic species producing song in the phylogenetic studies (see Table S1). *Agnotecous* species are known to produce sound above 9 kHz with a dominant frequency between 12 and 19 kHz, well above the 7 kHz threshold mentioned above [44]–[45]. The global acoustic method seems unable to detect the differences potentially due to the *Agnotecous* species as the distance value between sites was flat at this frequency range. This effect might be due to a frequency turnover from one *Agnotecous* species to another, on a similar frequency range. However, since most birds, Orthoptera and cicada species sing below 8 kHz, the threshold of biophony assessment in soundscape ecology [46], this could explain the between-site acoustic differences. New Caledonia counts 183 bird species including 23 regional endemics [47]. Due to their high dispersion level, birds could not be considered as narrow microendemics such as insects, lizards or plants species, limited to one small mountain. However, some bird species were described as being restricted to a part of the island as in the case of eleven species of birds inventoried in New Caledonia [48]–[49]. This could partially explain the between-site acoustic differences. Unfortunately, other singing groups (e.g., Orthoptera or cicadas) are too poorly known in the island and this prevents making detailed interpretations about the differences observed in this study.

As in the case of any other sampling methods, acoustic sampling method may be biased in some cases. For example, the distances between the targeted individuals and the sampling device (here the microphone), the vegetation structure or the individual behaviour (here the singing repertoire) could have an impact on the value calculated from the sample (here the acoustic indices). Massive sampling as the one done here (more than 6 000 samples) should, however, buffer such biases. As demonstrated in preliminary analyses, different background noises could generate significant differences between sites and lead to biased results. An appropriate processing of background noise is therefore decisive when undertaking acoustic comparisons. The question of acoustic bias for a rapid acoustic survey method should be estimated in controlled environments in further research programmes.

Overall, even if the global acoustic approach was limited to three sites with three points per site, it nevertheless has the potential of revealing compositional differences between sites and could therefore help in conservation management. Such a clear message from acoustics was not trivial as the sites showed a low level of ecological differentiation, and produced an equivalent acoustic activity level, under similar environmental parameters. These first results need to be supported by other study cases, but they open up new possibilities for biodiversity estimation and monitoring on a large scale. Numerous recorders could be deployed over a vast territory covering different sites and habitats. Such a global acoustic sampling could be achieved quite quickly and could be especially useful in hotspots where exploring biodiversity is an urgent task and where inventorying the diversity is very difficult because it has to rely on long-term sampling by experts. Microendemism is a well-identified challenge for local conservation policies, which requires an improvement in the number, size and quality of protected areas [9], [50]. Detecting dissimilarity and complementarity between sites could help in delimiting areas to conserve and the rapid acoustic survey would appear to be a pertinent approach as it is non-invasive and is a large-scale method.

## Supporting Information

Figure S1 **Acoustic differences between the ambient noises of the three sites.** The ambient noises coming from the different sites measured on files with null animal activity. **A**: Results of Principal Coordinate Analysis (PCoA) applied to the acoustic dissimilarities among recordings with factor “Site” as a supplementary variable subsequently projected on the map (A: Aoupinié, K: Koghis, M: Mandjélia). **B**: Results of PCoA applied to the acoustic dissimilarities among recordings, with factor “Hours” as a supplementary variable subsequently projected on the map. **C**: results of Distance-based ReDundancy Analysis (dbRDA) applied to the acoustic dissimilarities among recordings, with factor “Site” as an explanatory variable (A: Aoupinié, K: Koghis, M: Mandjélia). **D**: Results of dbRDA applied to the acoustic dissimilarities among recordings, with factor “Hour” as an explanatory variable. The dispersion ellipses surround the position of a site (Figure S1A) or of a time period (Figure S1B) providing an index of the dispersion of recording points around the site/time centroids (67% of recordings collected at a given time or at a site are expected to be in the associated ellipse).
(EPS)

Figure S2 **Acoustic differences between sites measured on a balanced sub-sample of hours.** Results of Distance-based ReDundancy Analysis (dbRDA) measured on a balanced sub-sample of hours, applied to the acoustic dissimilarities among recordings, with factor “Site” as an explanatory variable (A: Aoupinié, K: Koghis, M: Mandjélia).
(EPS)

Table S1 **Data describing the biodiversity of the three sites through 14 genera and 4 families.** Since these data come from phylogenies and inventories based on different geographical sampling, specimens are not sampled in every site. The main number determines the presence or absence of the taxa on the site, whereas the number in parenthesis determines whether the authors sampled on the site (1) or not (0).
(DOC)

Table S2 **Number and percentage of files associated to different noise types and number of files after exclusion of the noisy files for each site.**
(DOC)

Table S3 **Number and percentage of files with different activity levels for each site after the exclusion of noisy files.**
(DOC)
