# Supplementary material for: Biodiversity Sampling Using a Global Acoustic Approach: Contrasting Sites with Microendemics in New Caledonia
Source: PLoS One. 2013 May 29;8(5):e65311. doi: 10.1371/journal.pone.0065311 (PMC3667079; doi:10.1371/journal.pone.0065311)
Supplement: Table S1 — Data describing the biodiversity of the three sites through 14 genera and 4 families. Since these data come from phylogenies and inventories based on different geographical sampling, specimens are not sampled in every site. The main number determines the presence or absence of the taxa on the site, whereas the number in parenthesis determines whether the authors sampled on the site (1) or not (0). (DOC) [file pone.0065311.s003.doc]

**Table S1**. Data describing the biodiversity of the three sites through 14 genera and 4 families. Since these data come from phylogenies and inventories based on different geographical sampling, specimens are not sampled in every site. The number determine the presence or absence of the taxa on the site whereas the number in parenthesis determine if the authors sampled on the site (1) or not (0).

Taxa Aoupinié Mandjélia Koghis References

*Angustonicus* 1(1) 1(1) 1(1) Murienne 2006

*Lauraesilpha* 1(1) 1(1) 1(1) Murienne *et al*. 2008

*Tingidae* 1(1) 1(1) 1(1) Murienne *et al*. 2009

*Agnotecous* 1(1) 0(0) 1(1) Desutter-Grandcolas & Robillard 2006

*Xanthochorema* 0(1) 0(0) 1(1) Espeland *et al*. 2008

Hydropsychidae 0(0) 0(1) 1(1) Espeland & Johanson 2010a

*Agmina* 0(1) 0(1) 0(1) Espeland & Johanson 2010b

*Rhantus* 1(1) 1(1) 1(1) Balke *et al*.2009

*Troglosiro*  1(1) 0(0) 1(1) Sharma & Giribet 2009

Hydrobiidae 1(1) 0(1) 1(1) Haase & Bouchet 1998

*Rhacodactylus* 1(1) 0(1) 1(1) Good *et al*. 1997, Bauer *et al*. 1990

*Marmorosphax* 1(1) 1(1) 1(1) Sadlier *et al*. 2009

*Eurydactylodes* 1(1) 1(1) 0(1) Bauer *et al*. 2009

*Dierogekko* 0(1) 1(1) 0(1) Bauer *et al*. 2006

Scincidae 0(1) 0(1) 0(1) Sadlier *et al*. 2004

*Planchonella* 1(1) 1(1) 1(1) Swenson *et al*. 2007, Munzinger & Swenson 2009

Sapotaceae 1(1) 1(1) 1(1) Swenson *et al.* 2008, Munzinger & Swenson 2009, Swenson & Munzinger 2009, Swenson & Munzinger 2010a, b, c

*Diospyros* 1(1) 0(1) 1(1) Duangjai *et al*. 2009

Appendix S1 References

Balke M, Wewalka G, Alarie Y, Ribera I (2007) Molecular phylogeny of Pacific Island Colymbetinae: radiation of New Caledonian and Fijian species (Coleoptera, Dytiscidae). Zool Scripta 36: 173-200.

Bauer AM (1990) Phylogenetic systematics and biogeography of the Carphodactylini (Reptilia: Gekkonidae). Bonn Zoological Monographs 30: 1-220.

Bauer AM, Jackman T, Sadlier RA, Whitaker AH (2006) A Revision of the *Bavayia validiclavis* group (Squamata: Gekkota: Diplodactylidae), a Clade of New Caledonian Geckos Exhibiting Microendemism. Proc Calif Acad Sci 57: 503-547.

Bauer AM, Jackman T, Sadlier RA, Whitaker AH (2009) Review and phylogeny of the New Caledonian diplodactylid gekkotan genus Eurydactylodes Wermuth, 1965, with the description of a new species. In: Grandcolas P, editor. Zoologia Neocaledonica 7, Systematics and Biodiversity in New Caledonia. Mémoires du Muséum National d'Histoire Naturelle de Paris: 198, pp. 13-36.

Desutter-Grandcolas L, Robillard T (2006) Phylogenetic systematics and evolution of *Agnotecous* in New Caledonia (Orthoptera: Grylloidea, Eneopteridae). Syst Biol 31: 65-92.

Duangjai S, Samuel R, Munzinger J, Forest F, Wallnöfer B, Barfuss MH, Fisher G, Chase MW (2009) A multi-locus plastid phylogenetic analysis of the pantropical genus *Diospyros* (Ebenaceae), with an emphasis on the radiation and biogeographic origins of the New Caledonian endemic species. Mol Phylogenet Evol 52: 602–620.

Espeland M, Johanson KA, Hovmöller R (2008) Early *Xanthochorema* (Trichoptera, Insecta) radiations in New Caledonia originated on ultrabasic rocks. Mol Phylogenet Evol 48: 904-917.

Espeland M, Johanson KA (2010a) The effect of environmental diversification on species diversification in New Caledonian caddisflies (Insecta: Trichoptera: Hydropsychidae). J Biogeogr 37: 879-890.

Espeland M, Johanson KA (2010b) The diversity and radiation of the largest monophyletic animal group on New Caledonia (Trichoptera: Ecnomidae: *Agmina*). J Evolution Biol 23: 2112-2122.

Good DA, Bauer AM, Sadlier RA (1997) Allozyme evidence for the phylogeny of the giant New Caledonian geckos (Squamata: Diplodactylidae: *Rhacodactylus*), with comments on the status of R. leachianus henkeli. Aust J Zool 45: 317-330.

Haase M, Bouchet P (1998) Radiation of crenobiontic gastropods on an ancient continental island: the Hemistomia-clade in New Caledonia (Gastropoda: Hydrobiidae). Hydrobiologia 367: 43-129.

Munzinger J, Swenson U (2009) Three new species of *Planchonella* (Sapotaceae) with a dichotomous and an online key to the genus in New Caledonia. Adansonia 31: 175-189.

Murienne, J (2006) *Origine de la biodiversité en Nouvelle-Calédonie: Analyse phylogénétique de l’endémisme chez les Insectes Dictyoptères*, PhD Thesis, Université Pierre et Marie Curie - Paris 6.

Murienne J, Pellens R, Budinoff RB, Wheeler W, Grandcolas P (2008) Phylogenetic analysis of the endemic New Caledonian cockroach *Lauraesilpha*. Testing competing hypothesis of diversification. Cladistics 24: 802-812.

Murienne J, Guilbert E, Grandcolas P (2009) Species’ diversity in the New Caledonian endemic genera *Cephalidiosus* and *Nobarnus* (Insecta: Heteroptera: Tingidae), an approach using phylogeny and species’ distribution modelling. Biol J Linn Soc 97: 177–184.

Sadlier RA, Smith SA, Bauer AM, Whitaker AH (2009) Three new species of skink in the genus *Marmorosphax* Sadlier (Squamata: Sincidae) from New Caledonia. In Grandcolas P, editor. Zoologia Neocaledonica 7, Systematics and Biodiversity in New Caledonia. Mémoires du Muséum National d'Histoire Naturelle de Paris 198. pp. 247-263.

Sadlier RA, Smith SA, Bauer AM, Whitaker AH (2004) A new genus and species of live-bearing Scincid lizard (Reptilia: Scincidae) from New Caledonia. J Herpetol 38: 320-330.

Sharma P, Giribet G (2009) A relict in New Caledonia: phylogenetic relationships of the family Troglosironidae (Opiliones: Cyphophthalmi). Cladistics 25: 1-16.

Swenson U, Munzinger J, Bartish IV (2007a) Molecular phylogeny of *Planchonella* (Sapotaceae) and eight new species from New Caledonia. Taxon 56: 329-354.

Swenson U, Lowry II PP, Munzinger J, Rydin C, Bartish IV (2008) Phylogeny and generic limits in the Niemeyera complex of New Caledonian Sapotaceae: evidence of multiple origins of the anisomerous flower. Mol Phylogenet Evol 49: 909-929.

Swenson U, Munzinger J (2009) Revision of *Pycnandra* subgenus *Pycnandra* (Sapotaceae), a genus endemic to New Caledonia. Aust Syst Bot 22: 437–465.

Swenson U, Munzinger J (2010a) Revision of *Pycnandra* subgenus *Achradotypus* (Sapotaceae) with five new species from New Caledonia. Aust Syst Bot 23: 185-216.

Swenson U, Munzinger J (2010b) Revision of *Pycnandra* subgenus *Sebertia* (Sapotaceae) and a generic key to the family in New Caledonia. Adansonia 32, 239-249.

Swenson U, Munzinger J (2010c) Taxonomic revision of *Pycnandra* subgenus *Trouettia* (Sapotaceae) with six new species from New Caledonia. Aust Syst Bot 23**,** 333-370.
