# Supplementary material for: Biodiversity Sampling Using a Global Acoustic Approach: Contrasting Sites with Microendemics in New Caledonia
Source: PLoS One. 2013 May 29;8(5):e65311. doi: 10.1371/journal.pone.0065311 (PMC3667079; doi:10.1371/journal.pone.0065311)
Supplement: Table S2 — Number and percentage of files associated to different noise types and number of files after exclusion of the noisy files for each site. (DOC) [file pone.0065311.s004.doc]

**Table S2**. Number and percentage of files associated to different noise types and number of files after exclusion of the noisy files for each site.

Number of files Aoupinié Mandjélia Koghis

All 5708 4942 2441

With rain 2030 (35%) 632 (13%) 309 (13%)

With wind 572 (10%) 1894 (38%) 772 (31%)

With anthropogenic noise 98 (2%) 44 (1%) 169 (7%)

Without background noise 3008 (53%) 2372 (48%) 1191 (49%)
