# Supplementary material for: Biodiversity Sampling Using a Global Acoustic Approach: Contrasting Sites with Microendemics in New Caledonia
Source: PLoS One. 2013 May 29;8(5):e65311. doi: 10.1371/journal.pone.0065311 (PMC3667079; doi:10.1371/journal.pone.0065311)
Supplement: Table S3 — Number and percentage of files with different activity levels for each site after the exclusion of noisy files. (DOC) [file pone.0065311.s005.doc]

**Table S3**. Number and percentage of files with different activity levels for each site after the exclusion of noisy files.

Aoupinié Mandjélia Koghis

Medium to high activity (> 2 song types) 1369 (46%) 790 (34%) 597 (50%)

Low activity (1-2 song types) 1270 (42%) 960 (40%) 519 (44%)

Null activity (no song) 369 (12%) 622 (26%) 75 (6%)
